# Risk Perception, Perception of Collective Efficacy and Sleep Quality in Chinese Adults during COVID-19 Pandemic in Hong Kong: A Cross-Sectional Study

**DOI:** 10.3390/ijerph182111533

**Published:** 2021-11-03

**Authors:** Shiang-Yi Lin, Kevin Kien Hoa Chung

**Affiliations:** 1 Department of Early Childhood Education, The Education University of Hong Kong, Tai Po, Hong Kong, China; shiangyi.lin@gmail.com; 2 Division of Social Science, Hong Kong University of Science and Technology, Kowloon, Hong Kong, China; 3 Centre for Child and Family Science, The Education University of Hong Kong, Tai Po, Hong Kong, China

**Keywords:** COVID-19 pandemic, coronavirus, subjective sleep quality, risk perception, fear of infection, rumination, perception of collective coordinated defense, collective efficacy beliefs

## Abstract

Background: Only a few studies have studied the link between risk perception and sleep in the context of the COVID-19 pandemic. The purpose of our study is to propose and test a theoretical model to understand the relationships between COVID-19 risk appraisals—risk perception and perception of collective coordinated defense (PCCD) in particular—and subjective sleep quality in Chinese adults in Hong Kong during the COVID-19 pandemic. COVID-19-related fear and rumination were examined as potential mediators of the relationships. Methods: Data were collected using a self-report online questionnaire from a convenience sample of 224 Chinese adults during the fourth wave of the COVID-19 pandemic in Hong Kong. Results: Risk perception and PCCD were found to predict poor sleep quality. Mediation analysis showed that both fear and rumination mediated the relationship between risk perception and sleep quality, whereas only fear mediated the relationship between PCCD and sleep quality. The model was an excellent fit to the data and accounted for 44% of the variance in sleep quality in Chinese adults. This study indicated that both perception of high risks of contracting COVID-19 and anticipations of collective disease preventive efforts had adverse effects on subjective sleep quality via increasing COVID-19-related fear. Conclusions: These findings underscore the need for addressing sleep problems induced by psychological consequences of the pandemic. While policy makers often deliver public messaging campaigns that frame disease prevention as a collective goal, developing evidence-based coping strategies to combat COVID-19 adverse impacts on psychological health is equally important.

## 1. Introduction

The COVID-19 pandemic has developed into a severe global health challenge. In Hong Kong, despite the government’s stringent measures to stop disease spread (e.g., contact tracing, public gathering restrictions, and border controls and quarantines), the surge in infections continues to affect public health. This study was conducted in Hong Kong during the 4th wave of COVID-19 pandemic in January 2021. During the investigation period of this study, the Hong Kong authorities extended mandatory quarantines for new arrivals to 21 days due to the emergence of the SARS-COV-2 Alpha strain. Lockdowns were imposed in a few neighborhoods for screening suspected cases and conducting compulsory coronavirus testing of the residents. Despite the relatively low infection and death rates in Hong Kong (11,019 total confirmed cases and 200 total deaths as of 1 March 2021), research findings from earlier infectious disease epidemics (e.g., the severe acute respiratory syndrome, SARS) suggested that the residents still experienced anticipatory fear and anxiety during and after infectious disease outbreaks [1]. These uncertainties toward COVID-19, along with societal consequences of prolonged preventive efforts [2], may continue to be prevalent and affect sleep quality in the general public in Hong Kong.

Research on the current COVID-19 pandemic and other infectious disease epidemics indicated that such public health crises negatively affect sleep quality [3,4]. A recent meta-analysis has reported a prevalence of 32.3% in sleep disorders among the general population in 13 countries during the COVID-19 pandemic [4]. A study conducted in Hong Kong at the onset of the COVID-19 outbreak suggested that 38.3% of participants had poorer sleep quality relative to pre-outbreak levels [5]. Studies conducted among European adults also found that changes in sleep quality during the pandemic were related to the elevated levels of psychological distress, such as negative affect and worry [6,7]. The prevalent sleep problems in the COVID-19 pandemic warrant a systematic examination of how individual and collective perceptions of the pandemic development have contributed to COVID-19-related affective consequences and sleep quality.

Risk perception has been well-studied for its positive impacts on facilitating protective health behaviors and adherence to the governments’ behavioral guidelines to prevent disease spread [8,9]. The literature on risk perception suggests that risk perception consists of cognitive and affective components: the cognitive component refers to the perceived likelihood of oneself or others contracting the virus, whereas the affective component refers to experienced worries about the developing disease [8,10]. However, only a handful of studies have examined the link between COVID-19 risk perception and sleep quality: to our knowledge, all the previous investigations focused on solely medical staff or healthcare workers [11,12]. For instance, Yin et al. (2021) reported that medical staff who perceived higher risks of contracting COVID-19 at work also had poorer sleep quality [12]. Because sleep requires a loss of awareness, heightened risk perception in a public crisis may threaten people’s sense of safety and interfere their sleep. Grounded on these research findings, we expected that people’s perception of risks of being affected by COVID-19 would predict their sleep quality.

Prior work has shown that heightened risk perception, when disproportionate, increases fear and loss of control, which often lead to stress- or anxiety-related symptoms in response to COVID-19 [13,14,15]. Indeed, a recent study conducted among American adults with no history of COVID-19 infection showed that higher risk perception of contracting COVID-19 was related to greater fear of infection, which in turn predicted greater traumatic stress symptoms [15]. Furthermore, given the well documented link between stress and sleep, a study conducted among Bangladeshi adults during the current pandemic has shown that perceived stress over the virus and fears of being affected by COVID-19 predicted poorer sleep quality [16]. The findings summarized above about the link between COVID-19 risk perception, fear and sleep quality demonstrated great relevance to adults’ sleep problems, including an important source of adult sleep problems during the COVID-19 pandemic. Therefore, we aimed to investigate how COVID-19 risk perception and its effect on worsening sleep quality and further examine fear as a mediator between risk perception and sleep quality.

In addition, we examined negative thinking or rumination as a second mediator for the link between risk perception and sleep quality. Rumination is a maladaptive response to a stressor, characterized by repetitive, intrusive and negative thoughts related to stressful events [17,18]. Research indicated that intrusive negative thoughts during rumination increase cognitive arousals that prevent individuals from disengaging themselves from a stressor and thus interfere their sleep [19]. Recent studies conducted during the current pandemic also showed that rumination mediated the path from perceived stress to sleep quality [20,21]. Drawn on these findings, we predicted that rumination, along with fear of infection, would mediate between risk perception and sleep quality.

In public emergencies, people’s behaviors are likely to be affected not only by their perceptions of threats and risks but also by their observations of others’ behaviors. Research on the psychology of collective action in a natural disaster (e.g., an earthquake) indicated that the feeling of all being ‘in the same boat’ motivates people to participate in protective measures in order to achieve a common goal against a public crisis [22]. Collective efficacy, according to Bandura (1995), refers to “the beliefs in their capacities to organize and execute the courses of action required to manage prospective situations” [23]. In the present study, we focused on a task-specific form of perceived collective efficacy—perceived efficacies in coordinated action against disease spread [24]. Perceptions of collective coordinated defense (PCCD) encompass individuals’ perceptions of their own community’s efficacy to collectively engage in protective measures to reduce risks of infection by, for instance, mobilizing to distribute face masks or other supplies, or volunteering in community preventive measures (such as sanitizing public areas, or remaining wary of people who violate a mandatory quarantine).

Perceptions of collective efficacy in coordinated action have received little attention in the study context of the COVID-19 pandemic. Research on risk perception and coping beliefs among survivors of a natural hazard (e.g., a flooding event) suggests that perceptions of collective efficacy in coordinated action predicted an increase in risk perception and fear [23]. Babcicky and Seebauer have argued that this task-specific form of collective efficacy allows social cohesion to be transformed into task-specific collective actions, in that individuals who believe in group efficacy to protect themselves against a natural hazard also tend to be more aware of the local risk issues [22,24]. In line with this prediction, we reasoned that individuals who perceive their community’s coordinated defense as efficacious to prevent disease spread may be more psychologically attuned to the pandemic development and the operations of preventive and controlling measures against the disease than individuals who perceive low collective efficacy. As a high level of PCCD may signal that one should remain vigilant to and psychologically ready for combating the disease and taking corresponding collective action, we expected that higher PCCD would predict greater fear of infection and rumination, and that PCCD may further predict poor sleep quality through fear and rumination. As far as we know, no work has examined the link between PCCD and sleep quality, our analysis thus represents a novel test of whether perceptions of collective efforts to combat the disease predicted the worsening of sleep quality during the COVID-19 public emergencies.

Taken together, preexisting research studying the link between COVID-19 risk perception and sleep quality has focused solely on healthcare workers or medical staff [12,25]. This study was among the first to test the link between COVID-19 risk perception and sleep quality in the general population in Hong Kong, China. Moreover, and importantly, based on research findings with respect to collective efficacy in other contexts (e.g., a natural hazard) [24], we expected that PCCD would predict COVID-19-related affective consequences and sleep quality. Hence, this study has two main objectives—to examine the links between risk perceptions, PCCD and sleep quality, and to test fear and rumination as potential mediators between risk perceptions, PCCD and subjective sleep quality in Chinese adults in Hong Kong. We hypothesized that COVID-19 risk perception and PCCD would directly and indirectly predict sleep quality through fear and rumination.

## 2. Materials and Methods

### 2.1. Procedure and Participants

This cross-sectional correlational study was conducted among Chinese adults from 21 December 2020 and 15 January 2021, during the 4th wave of COVID-19 pandemic in Hong Kong. Adults aged 20 years or above, able to read the instructions in Chinese, and currently residing in Hong Kong were invited to participate in a web-based study about their perception of the COVID-19 pandemic and sleep quality. This research was approved by the Human Research Ethics Committee of the Education University of Hong Kong. The procedures used in this study adhere to the tenets of the Declaration of Helsinki. A link to the study was posted on the website of a research center of the university and advertised via Facebook to boost the response rate. The study link was also disseminated by the researchers’ professional networks. On average, participants took 21.87 min (SD = 7.65, range = 7.50–44.85 min) to complete the study. All participants provided informed consent regarding their participation in the study and publication of their data before proceeding to the study, and they received a supermarket coupon of $25 HKD (= $3.22 USD) as a token of appreciation. To ensure data quality, the online study embedded three attention checks that required participants to choose a specific answer, or not to choose any answer at all. Participants who failed all the checks (*N* = 16) were excluded from the analyses. The final sample consisted of 224 adult participants.

### 2.2. Measures

#### 2.2.1. Sleep Quality

The Adult Sleep-Awake Scale was administered to assess how often each sleep behavior occurred during the past month (e.g., “After waking up during the night, I roll over and go right back to sleep” and “After I fall asleep, but during the night, I toss and turn in bed”) [26]. The scale covers sleep behavior on five behavioral dimensions (i.e., going to bed, falling asleep, maintaining sleep, reinitiating sleep, and returning to wakefulness in the morning). Participants rated each item on a 6-point scale (1 = never to 6 = always) with respect to how frequently each behavior occurred. The scale showed good internal consistency (α = 0.89). A higher mean score indicates better sleep quality.

#### 2.2.2. Risk Perception

A 4-item scale was developed to measure the cognitive and affective components of risk perception [8,10]. The cognitive component consists of participants’ perceived *likelihood* of (1) being directly and personally affected by COVID-19 themselves, (2) their friends and family living in the same region (i.e., Hong Kong) being directly affected by COVID-19, and (3) many people living in the same region being affected in the next 3 months. Each statement was rated on a 5-point scale (1 = very unlikely to 5 = very likely). An additional item for assessing the affective component of risk perception captures the extent to which participants felt *worried* about the situations of COVID-19 pandemic. This statement was rated on a 5-point scale (1 = not worried at all to 5 = very worried). The scale showed good internal consistency (α = 0.85). A higher mean score indicates higher risk perception of the COVID-19 impact on oneself and others living in Hong Kong.

#### 2.2.3. Fear of Infection

Fear of infection was assessed with 8 items modified from Ho and colleagues’ (2005) SARS Fear Scale [27]. The items have been previously adapted to assess fear of infection and the feeling of loss of control over health or life due to COVID-19 (e.g., “I suspect whether I have been infected”, “I feel that life is threatened”) [13]. Participants rated the items on 4-point scale (1 = definitely false to 4 = definitely true). The scale showed excellent internal consistency (α = 0.91). A higher mean score indicates a greater level of fear of being infected and a stronger feeling of loss of control over health or life.

#### 2.2.4. Rumination

Rumination was assessed with Perseverative Thinking Questionnaire [17,18], which assessed the presence of and dwelling on worry and other negative thoughts during bedtime (e.g., “I think about many problems without solving any of them”, “my thoughts take up all my attention”). The 15 items were rated on a 5-point scale (1 = never to 5 = always). The scale showed excellent internal consistency (α = 0.97). A higher mean score indicates a higher frequency of rumination.

#### 2.2.5. Perception of Collective Coordinated Defense

This scale consists of five items adapted from Drury and colleagues’ (2016) scale that measured observed community coordination efforts [22]. The items assessed the extent to which participants observed situations where others in the community were involved in coordinated and collective defense to prevent the spread of COVID-19 (e.g., “people were involved in the community defense against the spread of COVID-19”). Participants rated each item on a 5-point scale (1 = strongly disagree to 5 = strongly agree). The scale showed excellent internal consistency (α = 0.90). A higher mean score indicates higher PCCD. A full list of the COVID-19-related measures and descriptive statistics is provided in Table 1.

### 2.3. Data Analyses

Data analyses were performed using SPSS Version 26 and R. Descriptive statistics were used to summarize demographic information, and Pearson’s correlations were performed to assess the associations between psychological antecedents (i.e., risk perception and PCCD), affective responses (i.e., fear and rumination), and sleep quality. Spearman’s ρ was used to examine the correlation between the study variables and demographic variables (i.e., age, gender, marital status, and education, employment status and household income levels).

To test our hypothesized model, a mediation analysis was conducted using the lavaan package in R. Parameters were estimated with maximum likelihood method. We expected both risk perception and PCCD to predict fear and rumination. Next, we expected fear and rumination to be directly related to sleep quality. Finally, we expected risk perception and PCCD to directly predict sleep quality, but also to have indirect effects on sleep quality via fear and rumination. Gender, age and marital status were entered as control variables in the model. The fit indices used to assess model fit are the comparative fit index (CFI), Tucker-Lewis Index (TLI), the root-mean-square error of approximation (RMSEA) and standardized root mean square residual (SRMR). Good model fit was determined by the cut-off of 0.95 value for CFI and TLI, 0.08 for the RMSEA and 0.06 for the SRMR [28]. The model used 1000 samples through 95% percentile bootstrap confidence intervals (CIs) to determine the indirect effects of the proposed mediators. The effect is considered as significant if the bootstrapped CI does not include 0.

## 3. Results

The sample consisted of 224 adults (187 female, 83.48%). The majority of participants (75.45%) aged below 40 years old. About half of the participants (50.89%) were single, while 45.98% of them were married and 3.13% were divorced or widowed. Description information about participants’ other demographic characteristics is shown in Table 2.

Table 3 presents descriptive statistics and bivariate associations among study variables. The normality of distribution of the data was examined by skewness and kurtosis. The skewness and kurtosis of the study variables were acceptable (|skewness| < 2, |kurtosis| < 7) [29]. As predicted, risk perception was positively correlated with fear and rumination, and negatively correlated with sleep quality. Similarly, PCCD was positively correlated with fear and rumination, and negatively associated with sleep quality. Furthermore, fear was weakly and positively associated with rumination. Both fear and rumination were negatively associated with sleep quality. Examination of the strength of bivariate correlations indicated that the variables were only moderately related with each other (the correlation coefficients were not higher than 0.60). Collinearity diagnostics showed that the variance inflation factors ranged from 1.09 to 1.63, suggesting that no potential multicollinearity existed in the data.

The examination of associations between demographic characteristics and study variables indicated that being female (vs. male) was related to higher risk perception (ρ = 0.161, *p* = 0.016), and being married (vs. single) was related to less rumination (ρ = −0.170, *p* = 0.011). None of the demographic characteristics were correlated with sleep quality (ρs < 0.084, *p*s > 0.210).

### Mediation Analyses

The proposed model had an excellent fit, CFI = 0.999, TLI = 0.988, RMSEA = 0.036, 90%CI [0.000, 0.186], SRMR = 0.014. When participant age, gender and marital status were entered as control variables, the inclusion of control variables did not cause any substantial changes in the estimates of the proposed relationships between variables, but resulted in a worse model fit, CFI = 0.685, TLI = 0.333, RMSEA = 0.174, 90%CI [0.148, 0.203], SRMR = 0.102. Table 4 summarizes the estimated coefficients of direct effects and bootstrapped 95% percentile confidence intervals. We predicted that fear and rumination mediated the relationship between risk perception and sleep quality. As shown in Table 4, the results indicated that risk perception was positively related to fear (*B* = 0.759, *SE* = 0.071, *p* < 0.001, 95% CI = [0.622, 0.899]) and rumination (*B* = 0.279, *SE* = 0.103, *p* = 0.007, 95% CI = [0.089, 0.501]), both of which were negatively associated with sleep quality (Fear: *B* = −0.189, *SE* = 0.053, *p* < 0.001, 95% CI = [−0.298, −0.091]; Rumination: *B* = −0.450, *SE* = 0.039, *p* < 0.001, 95% CI = [−0.525, −0.374]). The residual direct effect of risk perception on sleep quality was nonsignificant (*B* = 0.076, *SE* = 0.074, *p* = 0.302, 95% CI = [−0.073, 0.218]). Similarly, we found that PCCD, as expected, predicted greater fear (*B* = 0.196, *SE* = 0.069, *p* = 0.004, 95% CI = [.064, 0.334]) and rumination (*B* = 0.166, *SE* = 0.084, *p* = 0.048, 95% CI = [−0.001, 0.328]). The residual direct effect of PCCD on sleep quality was nonsignificant (*B* = −0.070, *SE* = 0.056, *p* = 0.208, 95% CI = [−0.174, 0.043]).

**Figure 1:**
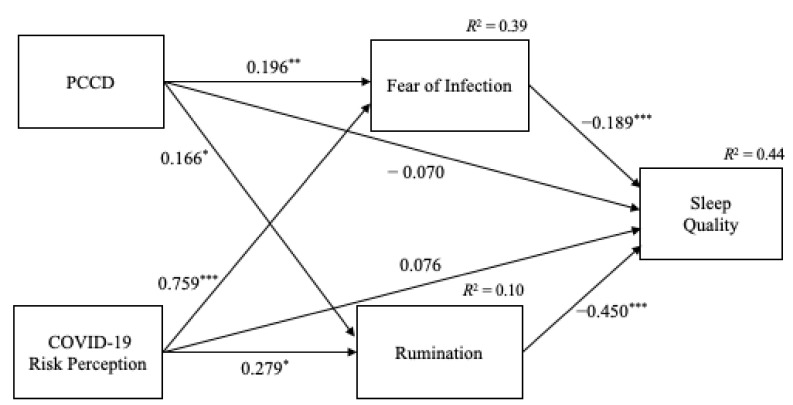
Mediation model. Note. The direct effect (standardized coefficients) of each path. * *p* < 0.05. ** *p* < 0.01. *** *p* < 0.001. Age, gender and marital status were entered as control variables (not shown in the figure).

The results of the indirect effects are shown in Table 5. Risk perception was indirectly related to sleep quality through fear of infection (*B* = −0.144, *SE* = 0.043, *p* = 0.001, 95%CI = [−0.240, −0.064]) and rumination (*B* = −0.125, *SE* = 0.047, *p* = 0.008, 95% CI = [−0.226, −0.039]). In addition, PCCD indirectly predicted sleep quality through fear (*B* = −0.037, *SE* = 0.017, *p* = 0.026, 95%CI = [−0.075, −0.010]) but not rumination (*B* = −0.075, *SE* = 0.039, *p* = 0.055, 95%CI = [−0.152, 0.001]). The model accounted for a total of 44% of the variance in sleep quality in Chinese adults. Figure 1 demonstrates the standardized coefficients of mediation results.

## 4. Discussion

The current COVID-19 situations have caused prevalent sleep problems. We tested a mediation model to examine the psychological factors that contribute to the worsening of sleep quality during the COVID-19 pandemic in Hong Kong. We also sought to examine whether fear and rumination mediated the relationships between risk perception, PCCD and sleep quality. After controlling for participant age, gender, and marital status, we found that risk perception had a direct effect on fear and rumination, suggesting that individuals who perceived higher risks of contracting COVID-19 also experienced greater fear and more frequent rumination than those who perceived lower risks. Both fear and rumination were negatively associated with sleep quality, meaning that fear of infection and rumination indeed contributed to worsen sleep quality during the pandemic. The analysis of indirect effects indicated that the relationship between risk perception and sleep quality was mediated by both fear and rumination. The results indicated that heightened COVID-19 risk perception predicted the worsening of sleep quality, both directly and indirectly, through increasing fear and rumination.

The tested pathway from risk perception, rumination to sleep quality corroborated previous research findings, which demonstrated that rumination mediated the relationship between perceived stress and sleep quality in college/university students from seven countries during the COVID-19 pandemic [20]. Although the present study related to individual and collective perceptions of risks, and Du and colleagues assessed perceived stress [20], the current findings resembled their results in that rumination mediated the route from perception of stressors to sleep quality.

This study also investigated whether individuals’ PCCD, perception of collective efficacy in coordinated defense against disease spread, would contribute to affective consequences of the COVID-19 and sleep quality. The results showed that PCCD positively predicted fear and rumination, suggesting that individuals perceiving higher collective coordinated defense also experienced greater fear and more frequent rumination than those perceiving lower collective coordinated defense. The mediation analysis indicated that PCCD was related to poor sleep quality only through increasing the fear of infection. The finding buttressed our hypothesis that PCCD plays a crucial role in intensifying COVID-19-related fear, such that individuals perceiving their community as highly efficacious to coordinate and defend against virus spread also experienced greater fear of infection or a loss of control over life or health than those perceiving their community as less efficacious.

The findings regarding PCCD are in line with past research showing that efficacy beliefs in collective coordinated action were associated with increased fear, whereas the other component of collective efficacy—mutual support—reduced fear [24]. In other words, social or mutual support provided by a close-knit social network helps individuals develop a sense of safety and psychological resilience, thus lowering fear and perception of risks [30]. Efficacy beliefs in collective coordinated defense, on the other hand, attune individuals to respond to a threat or stressor collectively, thus predicting greater fear of infection that potentially mobilizes people to exert continued efforts to prevent the spread of disease [31].

Taken together, because individuals respond to a disaster not as isolated individuals but as members of their social affiliated group (e.g., their country or community) [32], the results with respect to PCCD suggest that the extent to which individuals perceived efficacious collective defense against COVID-19 strengthens COVID-19-related fear. An important implication of the findings points to the crucial role of collective efficacy beliefs in sustaining individuals’ vigilance and psychological readiness to participate in collective action that advances the group goals (i.e., reducing the infection risks collectively) [33]. The important finding that PCCD exerts an indirect effect on sleep quality through increasing fear of infection underscores the negative consequences of framing or implementing disease preventive measures as a collective goal for sustained vigilance to the disease development as well as the worsening of sleep quality.

There are limitations to this study. First, direct exposure to COVID-19 was not measured and controlled for in this study when examining the study variables. For instance, we did not measure questions about whether participants knew anyone who has been diagnosed with COVID-19, and whether they were aware of any confirmed case in their neighborhood. However, the chance of having contact with patients diagnosed with COVID-19 in Hong Kong is relatively low (a total of 11,000 confirmed cases out of its 7.5 million population) compared to other countries. Past research showed that direct experience with the virus was a less important predictor for risk perception in countries with fewer confirmed cases [34]. Thus, we considered that this limitation was unlikely to affect the results presented above. Second, we did not ask participants about their mental health diagnosis, which could have made them more vulnerable to COVID-19-related stress. Future studies would benefit by including questions that assesses individuals’ mental health and testing if those with poor mental health are more negatively impacted by heightened individual and collective perceptions of COVID-19 threats and risks [13].

Third, this study used a convenience sampling method due to the difficulty to obtain a nationally representative sample via a web-based survey. Expectedly, we had more responses from adults aged below 40 years (about 75% in our study vs. 41% in the Hong Kong population) and from females (about 84% in our study vs. 54% of females in the Hong Kong population) [35]. We acknowledged that the findings obtained in this study are likely subjected to biases related to convenience sampling. Indeed, research has shown that elderly people had the highest mortality rate from the new coronavirus and thus they also perceived higher risks of severity about the consequences of contracting COVID-19 than their younger counterparts [36,37]. When it comes to the gender difference in risk perception and fear, prior work suggests that women experienced greater fear than men during the current pandemic [16]. In light of the skewed sex- and age-ratio of our data, generalization of the current findings to the Chinese population in Hong Kong should be made with caution.

Fourth, the subjective nature of self-report measures may have rendered the results affected by recall and measurement biases. Future research can use sleep diaries and actigraphy to assess sleep quality (i.e., objective sleep quality). Finally, the study was cross-sectional. Hence, the relationships between variables were correlational and should not imply any causal sequence. We acknowledged the relationships between emotion and sleep may be bidirectional [19]. For instance, poor sleep quality may also exacerbate experienced fear and risk perception. Furthermore, it is possible that individuals’ sleep behaviors may have been adapted to COVID-19 preventive measures (such as confinement and remote work policies) during the time of investigation [38]. Future research should employ a longitudinal design by comparing pre- vs. post-pandemic data to better understand how the changes in risk perception and sleep behavior unfold over time with the development of the pandemic.

## 5. Conclusions

In the face of unprecedented public health emergencies in the current pandemic, the present research findings highlighted the downside of individual and collective vigilance to the pandemic development for the worsening of sleep quality in Chinese adults in Hong Kong. As the COVID-19 pandemic is a prolonged stressful event, these results underscore important psychological processes that have contributed to facilitate protective behaviors to stop disease spread but adversely impacted sleep quality in adults amid the current global crisis.

## Figures and Tables

**Table 1:** Items of COVID-19-related measures and descriptive statistics.

Item	*N*	*Mean*	*SD*
*Risk Perception* (from 1 = very unlikely to 5 = very likely)			
How worried are you personally about the situations of COVID-19 pandemic?	224	4.009	0.826
How likely do you think you will be directly and personally affected by COVID-19 in the next 3 months?	224	4.179	0.822
How likely do you think your friends and family living in Hong Kong will be directly affected by COVID-19 in the next 3 months?	224	4.107	0.744
How much do you agree or disagree with the statement that COVID-19 will affect many people currently living in Hong Kong?	224	4.522	0.606
*Fear* (from 1 = definitely false to 4 = definitely true)			
Fear that I will be infected	224	3.897	0.915
Suspect whether I have been infected	224	3.036	1.062
Feel that the virus is very close to me, and the virus may invade my body anytime	224	3.540	1.041
Feel very unsafe about myself	224	3.558	0.996
Feel that life is threatened	224	3.165	1.126
Feel that I have lost control of life	224	3.143	1.147
I often think about death/dying	224	2.522	1.120
I worry about other health problems regarding myself	224	3.902	0.908
*Perception of Collective Coordinated Defense* (from 1 = strongly disagree to 5 = strongly agree)			
People organized to help others (such as distributing face masks or other supplies)	224	4.018	0.715
People cooperated with strangers in response to the disaster	224	3.799	0.798
People acted together against COVID-19	224	3.531	0.932
People actively participated in the community defense against COVID-19, such as sanitizing public areas or staying wary of others who did not wear face masks or violate a mandatory quarantine	224	3.594	0.918
People participated in coordinated rationing of supplies, such as face masks or hand sanitizers	224	3.719	0.796

Note. *N* = number of participants; *SD* = standard deviation. All items originally in English were presented in Chinese to the participants in the study.

**Table 2:** Demographic characteristics.

		*N*	%
Age			
	21–30	87	38.84
	31–40	82	36.61
	41–50	37	16.52
	51–60	11	4.91
	above 61	7	3.13
Gender			
	Male	37	16.52
	Female	187	83.48
Marital Status			
	Single	114	50.89
	Married	103	45.98
	Divorced or widowed	7	3.13
Education			
	High school and below	23	10.27
	College	34	15.18
	Bachelor’s degree	117	52.23
	Master’s degree	49	21.88
	Doctoral degree	1	0.45
Employment			
	Employed	161	71.88
	Unemployed or retired	12	5.36
	Housewives	29	12.95
	Students	19	8.48
	Others	3	1.34
Monthly household income (HKD)			
	<$30,000	72	32.14
	$30,001–$60,000	87	38.84
	>$60,000	60	26.79
	Prefer not to say	5	2.23

Note. HKD = Hong Kong dollars.

**Table 3:** Bivariate associations between study variables.

		1	2	3	4	5	*Mean*	*SD*	Skewness	Kurtosis
1	Risk Perception	-	0.199 **	0.594 ***	0.215 **	−0.185 **	4.204	0.617	−1.161	2.183
2	PCCD		-	0.271 ***	0.170 *	−0.202 **	3.732	0.669	−0.616	1.025
3	Fear			-	0.258 ***	−0.342 ***	3.345	0.823	−0.445	−0.035
4	Rumination				-	−0.620 ***	2.771	0.879	−0.087	−0.294
5	Sleep Quality					-	4.244	0.689	−0.268	−0.229

Note. *N* = 224. *SD* = standard deviation. PCCD = perception of collective coordinated defense. * *p* < 0.05. ** *p* < 0.01. *** *p* < 0.001.

**Table 4:** Direct effects.

Predictor	Dependent Variable	*B*	*SE*	*z*	*p*	95% CI (Lower, Upper)
Risk Perception	Fear of Infection	0.759	0.071	10.675	< 0.001	(0.622, 0.899)
PCCD	Fear of Infection	0.196	0.069	2.860	0.004	(0.064, 0.334)
Gender	Fear of Infection	−0.024	0.133	−0.177	0.860	(−0.291, 0.242)
Age	Fear of Infection	−0.068	0.098	−0.694	0.488	(−0.256, 0.126)
Marital Status	Fear of Infection	−0.062	0.094	−0.657	0.511	(−0.239, 0.122)
Risk Perception	Rumination	0.279	0.103	2.694	0.007	(0.089, 0.501)
PCCD	Rumination	0.166	0.084	1.975	0.048	(−0.001, 0.328)
Gender	Rumination	0.083	0.127	0.651	0.515	(−0.161, 0.348)
Age	Rumination	0.012	0.125	0.093	0.926	(−0.223, 0.258)
Marital Status	Rumination	−0.323	0.123	−2.627	0.009	(−0.569, −0.091)
Fear of Infection	Sleep Quality	−0.189	0.053	−3.599	< 0.001	(−0.298, −0.091)
Rumination	Sleep Quality	−0.450	0.039	−11.425	< 0.001	(−0.525, −0.374)
Risk Perception	Sleep Quality	0.076	0.074	1.032	0.302	(−0.073, 0.218)
PCCD	Sleep Quality	−0.070	0.056	−1.259	0.208	(−0.174, 0.043)
Gender	Sleep Quality	0.157	0.080	1.968	0.049	(−0.005, 0.316)
Age	Sleep Quality	0.024	0.085	0.279	0.780	(−0.133, 0.202)
Marital Status	Sleep Quality	−0.023	0.078	−0.293	0.769	(−0.173, 0.127)

Note. *N* = 224. *B* = unstandardized beta; *SE* = standard error; CI = confidence interval; PCCD = perception of collective coordinated defense. Participant age, gender, and marital status were included as control variables in the path analyses. Gender is coded as 0 = male, 1 = female. Age is coded as 0 = 41 years or above, 1 = 40 years or below. Marital status is coded as 0 = single, divorced, or widowed, 1 = married.

**Table 5:** Indirect effects.

Indirect Effect	*B*	*SE*	*z*	*p*	95% CI (Lower, Upper)
Risk Perception -> Fear of Infection -> Sleep Quality	−0.144	0.043	−3.336	0.001	(−0.240, −0.064)
Risk Perception -> Rumination -> Sleep Quality	−0.125	0.047	−2.645	0.008	(−0.226, −0.039)
PCCD -> Fear of Infection -> Sleep Quality	−0.037	0.017	−2.232	0.026	(−0.075, −0.010)
PCCD -> Rumination -> Sleep Quality	−0.075	0.039	−1.918	0.055	(−0.152, 0.001)

Note. *N* = 224. B = unstandardized beta; *SE* = standard error; CI = confidence interval; PCCD = perception of collective coordinated defense. Participant age, gender and marital status were included as control variables in the mediation analyses. A mediation effect was considered significant if the 95% CI excluded 0.

## Data Availability

Access to the dataset is available upon reasonable request to the corresponding author.
